# Outcomes Associated with Healthcare-Associated Respiratory Syncytial Virus in Children’s Hospitals

**DOI:** 10.1093/jpids/piae099

**Published:** 2024-10-05

**Authors:** Lisa Saiman, Susan E Coffin, Larry K Kociolek, Danielle M Zerr, Aaron M Milstone, Margaret L Aldrich, Celibell Y Vargas, Morgan A Zalot, Megan E Reyna, Amanda Adler, Danielle Koontz, Emily R Egbert, Jassour Alrikaby, Luis Alba, Sonia Gollerkeri, Madelyn Ruggieri, Lyn Finelli, Yoonyoung Choi

**Affiliations:** Department of Pediatrics, NewYork-Presbyterian Morgan Stanley Children’s Hospital, Columbia University Irving Medical Center, New York, New York, USA; Department of Pediatrics, Children’s Hospital of Philadelphia, Philadelphia, PA, Perelman School of Medicine at UPenn, Philadelphia, Pennsylvania, USA; Department of Pediatrics, Ann & Robert H. Lurie Children’s Hospital of Chicago, Chicago, Illinois, USA; Department of Pediatrics, Seattle Children’s Hospital, Seattle, Washington, USA; Department of Pediatrics, Johns Hopkins University School of Medicine, Baltimore, Maryland, USA; Department of Pediatrics, Children’s Hospital at Montefiore, Bronx, New York, USA; Department of Pediatrics, NewYork-Presbyterian Morgan Stanley Children’s Hospital, Columbia University Irving Medical Center, New York, New York, USA; Department of Pediatrics, Children’s Hospital of Philadelphia, Philadelphia, PA, Perelman School of Medicine at UPenn, Philadelphia, Pennsylvania, USA; Department of Pediatrics, Ann & Robert H. Lurie Children’s Hospital of Chicago, Chicago, Illinois, USA; Department of Pediatrics, Seattle Children’s Hospital, Seattle, Washington, USA; Department of Pediatrics, Johns Hopkins University School of Medicine, Baltimore, Maryland, USA; Department of Pediatrics, Johns Hopkins University School of Medicine, Baltimore, Maryland, USA; Department of Pediatrics, Children’s Hospital at Montefiore, Bronx, New York, USA; Department of Pediatrics, NewYork-Presbyterian Morgan Stanley Children’s Hospital, Columbia University Irving Medical Center, New York, New York, USA; Department of Pediatrics, NewYork-Presbyterian Morgan Stanley Children’s Hospital, Columbia University Irving Medical Center, New York, New York, USA; Center for Observational and Real-World Evidence, Rahway, New Jersey, USA; Center for Observational and Real-World Evidence, Rahway, New Jersey, USA; Center for Observational and Real-World Evidence, Rahway, New Jersey, USA

**Keywords:** escalation of respiratory support, healthcare-associated infections, respiratory syncytial virus

## Abstract

To determine if healthcare-associated (HA)-respiratory syncytial virus (RSV) is associated with worse outcomes, this multicenter cohort study studied 26 children with HA-RSV and 78 matched non-HA-RSV patients of whom 58% and 55%, respectively, had ≥2 comorbidities. Overall, 39% of HA-RSV versus 18% of non-HA-RSV patients required respiratory support escalation (adjusted odds ratio (aOR) 5.1, CI_95_ 1.4, 19.1).

## INTRODUCTION

Healthcare-associated (HA) respiratory syncytial virus (HA-RSV) infection is associated with morbidity and occasional mortality, especially in children with chronic conditions [1, 2]. However, our understanding of adverse outcomes associated with HA-RSV infections has been limited due to the lack of a control group with which to compare outcomes. Thus, we compared risk factors and outcomes in children with HA-RSV infections versus hospitalized children without HA-RSV infections. We hypothesized that HA-RSV infections are independently associated with worse outcomes.

## METHODS

As determined by the study protocol, we performed a multi-center prospective cohort study of children with HA-RSV infections from October 2020 to April 2022 identified at 6 academic children’s hospitals in the United States. Sites have a median of 309 beds (interquartile range, [IQR] 197, 453), intensive care units (ICUs), neonatal ICUs, and pediatric emergency departments. All perform multiplex polymerase chain reaction assays for respiratory pathogens, which detect RSV, in children with respiratory symptoms on admission and those who develop new-onset respiratory symptoms while hospitalized. Each site obtained institutional review board approval or exemption to conduct this study with a waiver of informed consent.

### Eligible and Ineligible HA-RSV and Non-HA-RSV Patients

Cases of HA-RSV infections in children ≤18 years old were defined as new-onset or worsening respiratory symptoms and positive RSV test was obtained ≥72 hours after admission [3]. To ensure full case capture, sites also reviewed respiratory viral test results from their clinical microbiology laboratories to identify additional children who met the case definition.

Children were ineligible to be selected as patients with HA-RSV if they had positive RSV tests within the past 6 months, HA-RSV infection was diagnosed at an outside hospital prior to transfer to study sites, or respiratory symptoms were present on admission, but testing was not performed within 72 hours of admission.

Sites identified all potential non-HA-RSV patients admitted within 2 months of each patient with HA-RSV’s day 0, defined as the day of the positive RSV test. Three non-HA-RSV patients were matched to each patient with HA-RSV by study site, age on admission, and length of stay (LOS) at least as long until day 0 for each patient with HA-RSV. For patients with HA-RSV with LOS >3 months, we required that matched non-HA-RSV patients had LOS at least >3 months and day 0 of observation was assigned to be 90 days. Matching on LOS prior to RSV infection provided a similar time for potential RSV exposure in HA- and non-HA-RSV patients and provided a potential surrogate for the severity of illness (Supplementary Figure 1). We used a standardized delta approach that equally weighted age and LOS (Supplementary Table 1). Non-HA-RSV patients were ineligible for selection if they were hospitalized for community-acquired (CA)-RSV or had a positive RSV test within the past 6 months.

### Study Outcomes

We compared the demographic and clinical characteristics of HA-RSV and non-HA-RSV patients. We evaluated selected outcomes temporally associated with HA-RSV in patients with HA-RSV with these outcomes during the non-HA-RSV patients’ hospitalizations (Supplementary Figure 1). The primary outcome was an escalation of respiratory support on 1 or more days from day –2 to day +4. Escalation was defined as a change from room air to supplemental oxygen, an increase in the fraction of inspired oxygen (FiO_2_) on the same respiratory support modality, a change from noninvasive to invasive respiratory support, or an increase in FiO_2_ on the same invasive modality [3]. Noninvasive modalities included nasal cannula oxygen, high-flow oxygen, continuous positive airway pressure, or bi-level positive airway pressure (BiPAP). Invasive modalities included mechanical ventilation or extracorporeal membrane oxygenation (ECMO). Additional outcomes included transfer to the pediatric ICU (PICU) on 1 or more days from day –2 to day +4 and in-hospital mortality.

### Data Collection and Analysis

Collected data included comorbid conditions [4], respiratory support within the 7 days prior to hospitalization, co-detection of other viruses detected from patients with HA-RSV on day 0, and positive blood and respiratory cultures obtained from day –2 to day +2.

We conducted unadjusted bivariate analyses using conditional logistic regression to compare demographic and clinical factors in HA-RSV vs. non-HA-RSV patients. Factors significantly different in HA-RSV vs. non-HA-RSV patients (*P*-values < .10) were assessed in a multivariable conditional logistic regression model.

To assess the escalation of respiratory support, we conducted bivariate conditional logistic regression that accounted for matching. Then we conducted a multivariable conditional logistic regression that included the factors significantly different between HA-RSV and non-HA-RSV patients in the bivariate analysis. The regression models did not include adjustments for site-level clustering, but the site was considered by the matching design. A Wilcoxon signed-rank test compared the median number of days spent in the PICU by HA-RSV vs. non-HA-RSV patients in those transferred to the PICU. Analyses were completed using Stata/BE 17.0 for Mac.

## RESULTS

We identified 26 patients with HA-RSV detected on a median of the seventh day of hospitalization (IQR 5, 18 days) and selected 78 non-HA-RSV patients. The sex and age distributions (Table 1) and reasons for hospital admission (Supplementary Table 2) among HA-RSV and non-HA-RSV patients were similar. Overall, 57.7% and 55.2% of HA-RSV and non-HA-RSV patients, respectively, had ≥2 comorbid conditions. Patients with HA-RSV were more likely to have cardiovascular comorbid conditions (*P* = .005) and less likely to have respiratory comorbid conditions (*P* = .02). During the week prior to day –2, 33 and 17 patients were hospitalized in the PICU and NICU, respectively.

**Table 1.** Demographic and Selected Clinical Characteristics and Comorbid Conditions of Children with and without HA-RSV Infections

Characteristics		All *N* = 104	Children with HA-RSV *n* = 26	Children without HA-RSV *n* = 78	*P*-value Bi-variate	*P*-value Multi-variate analysis^a^
Demographic characteristics						
Male sex, *n* (%)		43 (41.3%)	13 (50%)	40 (51.3%)	.91	-
Median age, months (IQR)		15.3 (3.3, 44.9)	15.0 (4.0, 51.0)	15.3 (3.0, 41.0)	.73	-
Age, *n* (%)						
	≤12 months	47 (45.2)	11 (42.3%)	36 (46.2%)	.99	–
	13–60 months	43 (41.3)	11 (42.3%)	32 (41.0%)	.99	–
	>60 months	14 (13.5)	4 (15.4%)	10 (12.8%)	Ref	–
Race, *n* (%)						
	White	41 (39.4)	5 (19.2%)	36 (46.2%)	Ref	Ref
	Black/African American	13 (12.5)	5 (19.2%)	8 (10.3%)	**.02**	**0.06**
	Asian	6 (5.8)	0 (0%)	6 (7.7%)	–	–
	American Indian, Alaska Native	1 (1.0)	0 (0%)	1 (1.3%)	–	–
	Other	32 (30.8)	12 (46.2%)	20 (25.6%)	**.02**	**0.04**
	Unknown	11 (10.6)	4 (15.4%)	7 (9.0%)	**.07**	0.12
Ethnicity, *n* (%)	Hispanic	38 (36.5)	9 (34.6%)	29 (37.2%)	Ref	–
	Non-Hispanic	56 (36.5)	14 (53.9%)	42 (53.9%)	.85	–
	Unknown	1 0(10.6)	3 (11.5%)	7 (9.0%)	.64	–
Insurance, *n* (%)						
	Private/parent employer/union	29 (27.9)	3 (11.5%)	26 (33.3%)	**.05**	0.12
	Other, including medicaid	73 (70.2)	22 (84.6%)	51 (65.4%)	Ref	–
	Uninsured/other/ unknown	2 (1.9)	1 (3.9%)	1 (1.3%)	.50	
**Selected clinical characteristics**						
Receiving respiratory support within 7 days prior to admission^b^		11/87 (12.6%)	4/23 (17.3%)	7/64 (10.9%)	.52	
Hospitalized in PICU week prior to day –2^c^		33 (29.8%)	6 (23.1%)	27 (34.6%)	.26	
Hospitalized in NICU week prior to day –2^c^		17 (16.3%)	3 (11.5%)	14 (17.9%)	.34	
**Comorbid conditions, *n* (%)**						
	Cardiovascular	35 (33.7)	14 (53.9%)	21 (26.9%)	**.01**	**0.005**
	Gastrointestinal	37 (35.6)	8 (30.8%)	29 (37.2%)	.54	–
	Neurologic/ Neuromuscular	28 (26.9)	6 (23.1%)	22 (28.2%)	.61	–
	Respiratory	28 (26.9)	3 (11.5%)	25 (32.1)	**.05**	**0.02**
	Other congenital/ Genetic defect	9 (8.7)	2 (7.7%)	7 (9.0%)	.85	–
	Premature/neonatal	34 (32.7)	7 (26.9%)	27 (34.6%)	.45	–
	Hematologic/ Immunologic/rheumatologic	10 (9.6)	5 (19.2%)	5 (6.4%)	**.07**	0.42
	Malignancy	12 (11.5)	3 (11.5%)	9 (11.5%)	1.00	–
	Renal/urologic	18 (17.3)	6 (23.1%)	12 (15.4%)	.38	–
	Metabolic	10 (9.6)	1 (3.9%)	9 (11.5%)	.25	–
	Miscellaneous	2 (1.9)	0 (0%)	2 (2.3%)	–	–
**Number of comorbidities, *n* (%)**						
	0	9 (8.7)	2 (7.7%)	7 (9.0%)	Ref	–
	1	37 (35.6)	9 (34.6%)	28 (35.9%)	.97	–
	2–3	36 (34.6)	11 (42.3%)	25 (32.1%)	.72	–
	≥4	22 (21.2%)	4 (15.4%)	18 (23.1%)	.65	–

^a^Bolded *P*-values represent outcomes with *P*-values < .10.

^b^Excludes 17 children admitted > 3 months prior to day 0 or hospitalized in NICU.

^c^Day –9 to day –3.

Escalation of respiratory support was more frequent in patients with HA-RSV than non-HA-RSV patients (38.5% vs. 18.0%, respectively) (Table 2). When adjusted for comorbid conditions, escalation of respiratory support was 5.1 times (CI_95_ 1.4, 19.1) more likely in patients with HA-RSV. Transfer to the PICU was similar among HA-RSV (*n* = 5, 19.2%) vs. non-HA-RSV patients (*n* = 9, 11.5%; *P* = .44). Zero patients with HA-RSV and two non-HA-RSV patients died during hospitalization.

**Table 2.** Clinical Outcomes Associated With HA-RSV in Children

Outcome (*n*, %)	Children With HA-RSV *n* = 26	Children Without HA-RSV *n* = 78	Odds Ratio (95% CI)	aOR^f^ (95% CI)
Escalation of respiratory support (any)	10 (38.5%)	14 (18.0%)	3.5 (1.1, 10.7)	5.1 (1.4, 19.1)
**Type of escalation of respiratory support**				Not applicable
• None	16 (61.5%)	64 (82.1%)	Ref	
• Change from room air to supplemental oxygen	4 (15.4%)	2 (2.6%)	15.8 (1.4, 174.4)	
• Increase in FiO_2_ on noninvasive modality^a^	4 (15.4%)	4 (5.1%)	6.21 (1.0, 38.8)	
• Change from noninvasive to invasive modality^b^	1 (3.8%)	3 (3.8%)	1.14 (0.1, 11.8)	
• Increase in FiO_2_ on invasive modality	1 (3.8%)	5 (6.4%)	0.66 (0.07, 6.6)	
Transfer to pediatric ICU^c^	5/18 (27.8%)	9/47 (19.1%)	0.45^d^	
If transferred, median days in ICU (IQR)	8 (8, 9)	4 (3, 4)	*P* = .21^e^	
In-hospital mortality	0 (0%)	2 (2.6%)	–	

^a^Noninvasive modalities included nasal cannula oxygen, high flow oxygen, continuous positive airway pressure, or bi-level positive airway pressure.

^b^Invasive modalities included mechanical ventilation or extracorporeal membrane oxygenation.

^c^Number transferred/number not in PICU on day –3. Any day transferred from day –2 to day +4.

^d^Chi-squre performed.

^e^Wilcoxon signed-rank test performed.

^f^Multivariable conditional logistic regression included respiratory and cardiac comorbid conditions significantly different between HA-RSV and non-HA-RSV patients (Table 1).

Adenovirus, human metapneumovirus, influenza A H3, or rhinovirus/enterovirus (*n* = 2) were co-detected in 5 (19%) patients with HA-RSV. Three HA-RSV and zero non-HA-RSV patients had positive blood cultures for pathogenic organisms, and none had positive respiratory cultures.

## DISCUSSION

We confirmed our hypothesis that children with HA-RSV infections were more likely to require escalation of respiratory support compared with matched non-HA-RSV patients. The impact of HA-RSV is notable as more than half of patients had ≥2 comorbid conditions, many of which are associated with increased risk of severe RSV infections.

HA-RSV outbreaks are described worldwide [5–7], but the burden of HA-RSV is likely underestimated, particularly in low- and middle-income countries, due to differing testing practices and decreased recognition of sporadic HA-RSV. Compared to community-acquired (CA)-RSV, HA-RSV can result in increased ICU admissions, mechanical ventilation, and cost [7, 8]. Furthermore, HA-RSV is responsible for 20% of RSV-related in-hospital deaths in lower- and middle-income countries [9]. However, adverse outcomes associated with HA-RSV are confounded by the underlying risk of severe RSV illness; children at risk for HA-RSV generally have more comorbid conditions than children with CA-RSV. To address the issue of confounding, we evaluated clinical outcomes attributable to HA-RSV using matched comparators without RSV who were themselves medically complex as 11% required respiratory support prior to admission, 55% had ≥2 comorbid conditions, and 55% were hospitalized in the PICU or NICU. After adjusting for such confounders, we determined that HA-RSV was an independent predictor for escalation of respiratory support.

Preventing HA-RSV infections requires infection prevention and control strategies including: avoiding presenteeism, (ie healthcare workers (HCW) working while ill), overcrowding, and understaffing; appropriate hand hygiene and personal protective equipment (masks and eye protection); cleaning and disinfection of environmental surfaces and patient-care equipment; improving ventilation and hospital design; maintaining a high index of suspicion for HA viral infections, prompt testing, and isolation [10–12]. The impact of new preventive strategies, such as nirsevimab and maternal RSVpreF vaccine, on HA-RSV infections is unknown.

This study had limitations. Sites were academic children’s hospitals, which may limit the study’s generalizability. Residual confounding related to underlying comorbid conditions, medical device use, and prolonged hospitalizations likely occurred. We did not collect treatment data for palivizumab. It is possible that minor respiratory symptoms, present either on admission or within the first 72 hours of admission were not noticed or documented, resulting in CA-RSV being misclassified as HA-RSV. Management of respiratory support was not standardized. Due to the small sample size, differences between rare outcomes such as PICU admission or death may not have been detected. Because we used LOS in the matching strategy, we could not compare the total LOS among HA-RSV and non-HA-RSV patients. The impact of viral co-detection and bloodstream infections on outcomes is uncertain. The study was conducted during the COVID-19 pandemic, which substantially impacted RSV epidemiology and recommendations for personal protective equipment. As we studied sporadic HA-RSV, our findings may not be applicable to HA-RSV outbreaks.

In conclusion, HA-RSV infection was an independent factor associated with the escalation of respiratory support. Strategies to reduce HA respiratory viral infections in children as well as standardizing case definitions and surveillance strategies are needed for HA-RSV and other respiratory viral pathogens.

## Supplementary Data

Supplementary materials are available at the *Journal of The Pediatric Infectious Diseases Society* online (http://jpids.oxfordjournals.org).

piae099_suppl_Supplementary_Figure_S1_Tables_S1-S2

## Notes

***Financial support.*** This work was supported by Merck Sharp & Dohme LLC, a subsidiary of Merck & Co., Inc., Rahway, NJ, USA.

***Potential conflicts of interest.*** All authors received grant funding provided to their institutions from Merck Sharp & Dohme LLC, a subsidiary of Merck & Co., Inc., Rahway, NJ, USA to perform this work. Madelyn Ruggieri MS, Lyn Finelli DrPH MS, and Yoonyoung Choi PhD MS RPh are employees of Merck Sharp & Dohme LLC, a subsidiary of Merck & Co., Inc., Rahway, NJ, USA. Lisa Saiman, MD, MPH, served on an advisory board and on a data safety monitory board for Merck & Co., Inc., Rahway, NJ, USA. Danielle Zerr MD MPH is a consultant for Allovir on endpoint adjudication committees for clinical trials.
