# Supplementary material for: Outcomes Associated with Healthcare-Associated Respiratory Syncytial Virus in Children’s Hospitals
Source: J Pediatric Infect Dis Soc. 2024 Oct 5;13(11):594–8. doi: 10.1093/jpids/piae099 (PMC11599150; doi:10.1093/jpids/piae099)
Supplement: piae099_suppl_Supplementary_Figure_S1_Tables_S1-S2 [file piae099_suppl_supplementary_figure_s1_tables_s1-s2.docx]

**Appendix:**

**Supplemental Figure 1: Matching strategy for HA-RSV and non-HA-RSV.** At each site, we identified non-HA-RSV patients who were admitted within 2 months of their respective HA-RSV patient's Day 0 (red dot). Three non-HA-RSV patients were selected for each HA-RSV patient based on the closest match by age on admission and length of stay (LOS). For the LOS match, we considered the HA-RSV patient's LOS before Day 0 and the non-HA-RSV patient's overall LOS (indicated by the red brackets). The primary outcome of escalation of respiratory support (indicated by the orange "**x**") was evaluated from Day -2 to Day +4. The gray dot indicates the day of discharge.

**Supplemental Figure 1.**

**
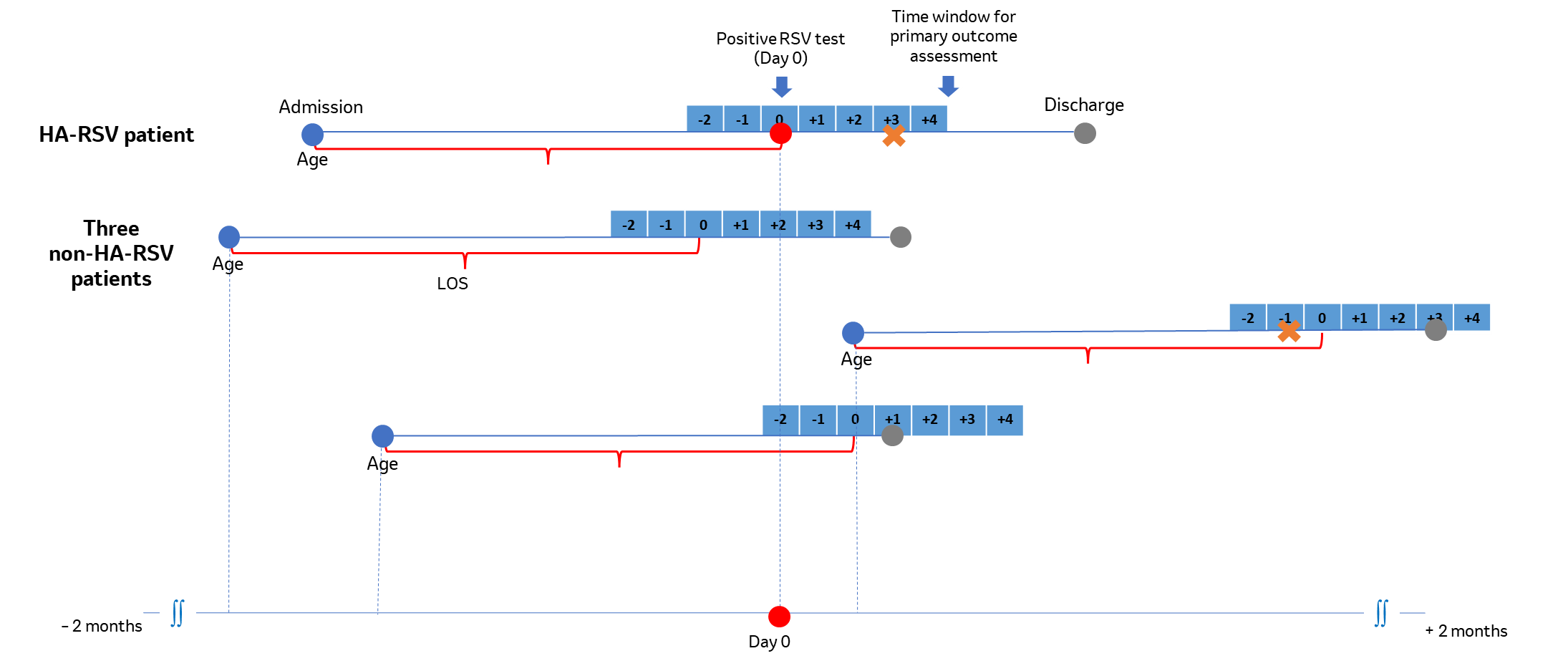
**

**Supplemental Table 1. Example of Standardized Delta Approach to Identify non-HA-RSV Patients^1^**

| **HA-RSV Patients** | | **Non-HA-RSV Patients** | |  |  |  |
| --- | --- | --- | --- | --- | --- | --- |
| **LOS by Day 0 (days)** | **Age (days)** | **LOS**  **(days)** | **Age**  **(days)** | **Standardized delta for LOS** | **Standardized delta for Age** | **Sum** |
| 468 | 1709 | 165 | 1733 | **I**(165-468)/468**I**=0.65 | **I**(1733-1709)/1709**I**=0.01 | 0.66 |
|  |  | 451 | 478 | **I**(451-468)/468**I**=0.04 | **I**(478-1709)/1709**I**=0.72 | 0.76 |
|  |  | 74 | 1650 | **I**(74-468)/468**I**=0.84 | **I**(1650-1709)/1709**I**=0.03 | 0.88 |

**^1^** Non-HA-RSV Patient matches were identified by equally weighting age and LOS. Standardized deltas for LOS and age were each calculated as the absolute difference between the HA-RSV patient and selected non-HA-RSV patients and divided by the HA-RSV patient’s values. The closest matches were the lowest sum of standardized delta values.

**Supplemental Table 2: Reasons for Hospitalization for HA-RSV and non-HA-RSV Patients**

| **Reason for admission** | **HA-RSV Patients**  **n=26** | **Non-HA-RSV Patients**  **n=78** |
| --- | --- | --- |
| Gastrointestinal | 3 (11.5%) | 14 (17.9%) |
| Respiratory distress/ failure | 4 (15.4%) | 11 (14.1%) |
| Prematurity | 1 (3.8%) | 12 (15.4%) |
| Cardiac | 4 (15.4%) | 5 (6.4%) |
| Transplant | 2 (7.7%) | 6 (7.7%) |
| Infection | 4 (15.4%) | 3 (3.8%) |
| Fever | 1 (3.8%) | 5 (6.4%) |
| Neurologic/ neurosurgical | 1 (3.8%) | 6 (7.6%) |
| Surgical procedure | 2 (7.6%) | 4 (5.1%) |
| Malignancy | 3 (11.5%) | 3 (3.8%) |
| Renal | 0 (0%) | 2 (2.6%) |
| Other^1^ | 1 (3.8%) | 7 (9%) |

**^1^** Other included trisomy 21 syndrome, small for gestational age, well-baby nursery admission, facial swelling, conjoined twins, joint swelling/ pain, fussy/weak/developmental regression, vaso-occlusive crisis
